# Supplementary material for: Bicontinuous Gyroid Phase of a Water-Swollen Wedge-Shaped Amphiphile: Studies with In-Situ Grazing-Incidence X-ray Scattering and Atomic Force Microscopy
Source: Materials (Basel). 2021 May 28;14(11):2892. doi: 10.3390/ma14112892 (PMC8198821; doi:10.3390/ma14112892)
Supplement: Supplementary file 1 [file materials-14-02892-s001.zip › materials-1235608-supplementary.pdf]

Supplementary Materials for

Bicontinious gyroid phase of a water-swollen wedge-shaped amphiphile: studies with in-situ grazing-incidence X-ray scattering and atomic force microscopy

Kseniia N. Grafskaia [1,2], Azaliia F. Akhkiamova [2,3], Dmitry V. Vashurkin [2,5], Denis S. Kotlyarskiy [2,5], Diego Pontoni [3], Denis V. Anokhin [1,2], Xiaomin Zhu [6], Dimitri A. Ivanov [1,2,4,5*]

[1] Moscow Institute of Physics and Technology, Instituskiy per. 9, Dolgoprudny, 141700, Russian Federation;

[2] Institute of Problems of Chemical Physics, Russian Academy of Sciences, Chernogolovka, Moscow Region, 142432, Russian Federation;

[3] Partnership for Soft Condensed Matter (PSCM), ESRF – The European Synchrotron, 71 avenue des Martyrs, 38043 Grenoble, France;

[4] Institut de Sciences des Matériaux de Mulhouse-IS2M, CNRS UMR 7361, Jean Starcky, 15, F-68057 Mulhouse, France

[5] Faculty of Chemistry, Lomonosov Moscow State University (MSU), GSP-1, 1-3 Leninskiye Gory, 119991 Moscow, Russia

[6] DWI – Leibniz-Institute for Interactive Materials e.V. and Institute for Technical and Macromolecular Chemistry of RWTH Aachen University, Forkenbeckstr. 50, D-52056, Aachen, Germany

* Correspondence: dimitri.ivanov@uha.fr

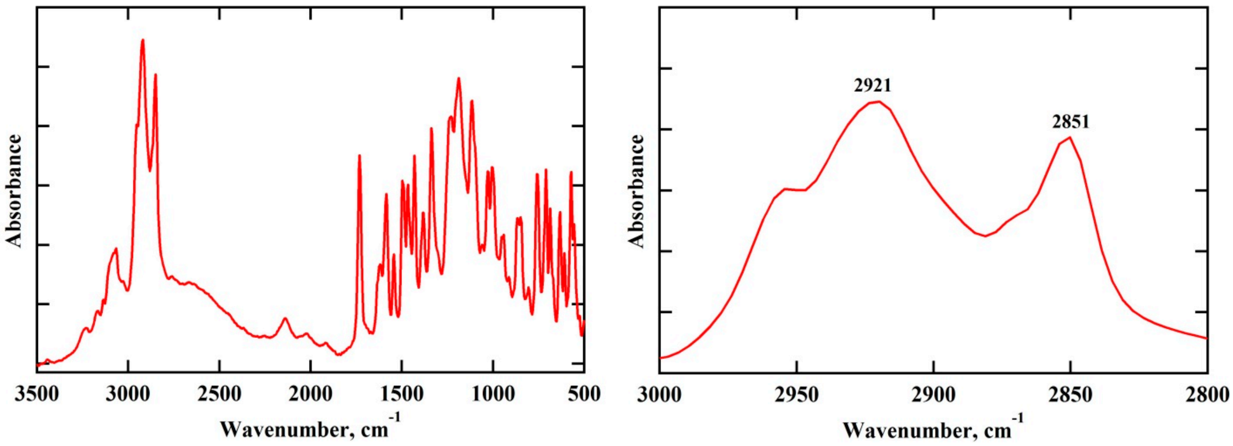

(a) (b)

Figure S1. (a) FTIR spectrum of C8AzoPyr powder after long-term storage at room temperature; (b) magnified region of (a) with anti-symmetric and symmetric stretching vibration modes of CH_2 groups.

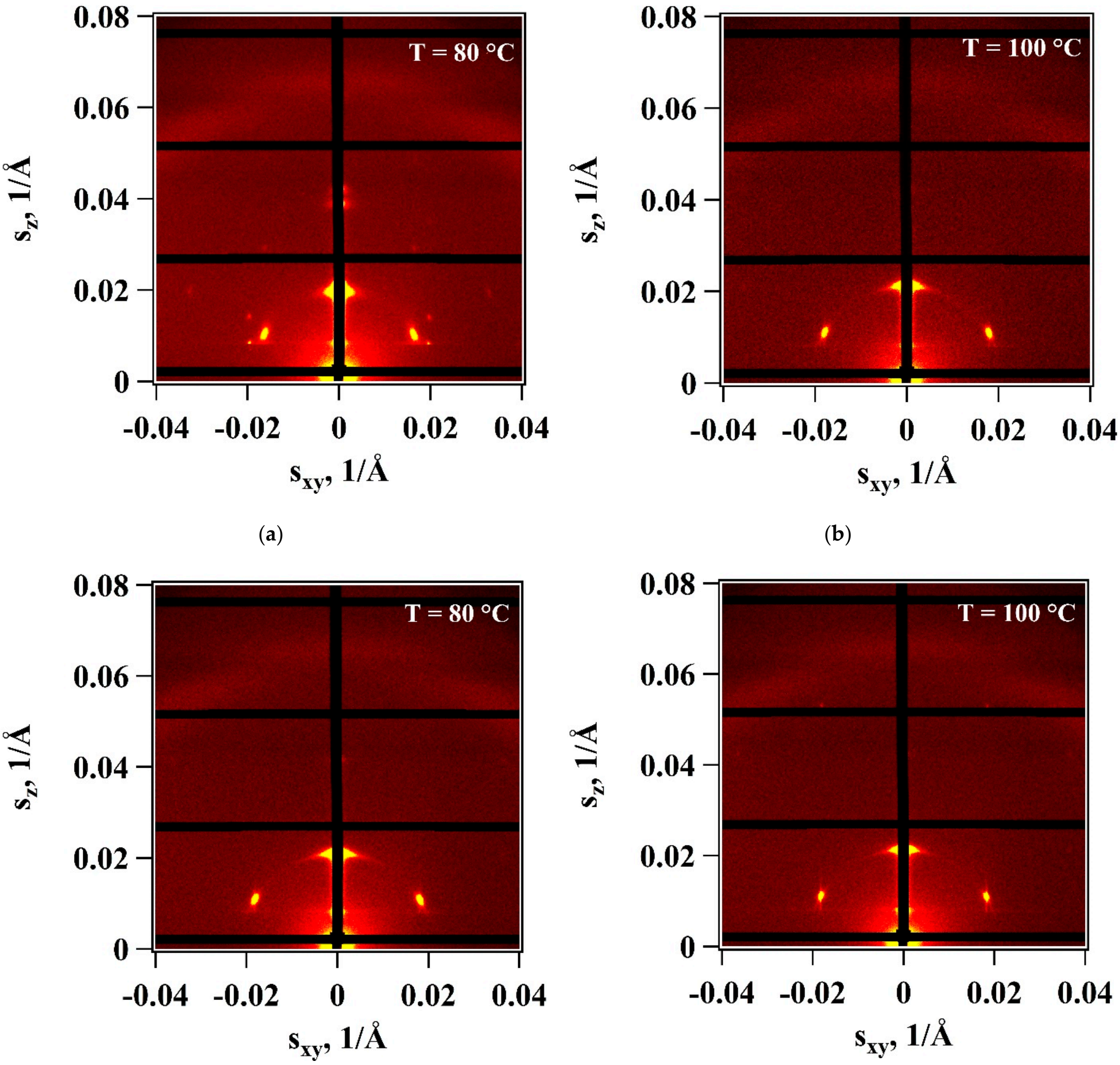

(a) (b)

(c) (d)

Figure S2. 2D GISAXS patterns of a dry C8AzoPyr thin film recorded during first heating from 25 ºC at 80 ºC (a) and at 100 ºC (b), and during second heating from 25 ºC at 80 ºC (c) and at 100 ºC(d).

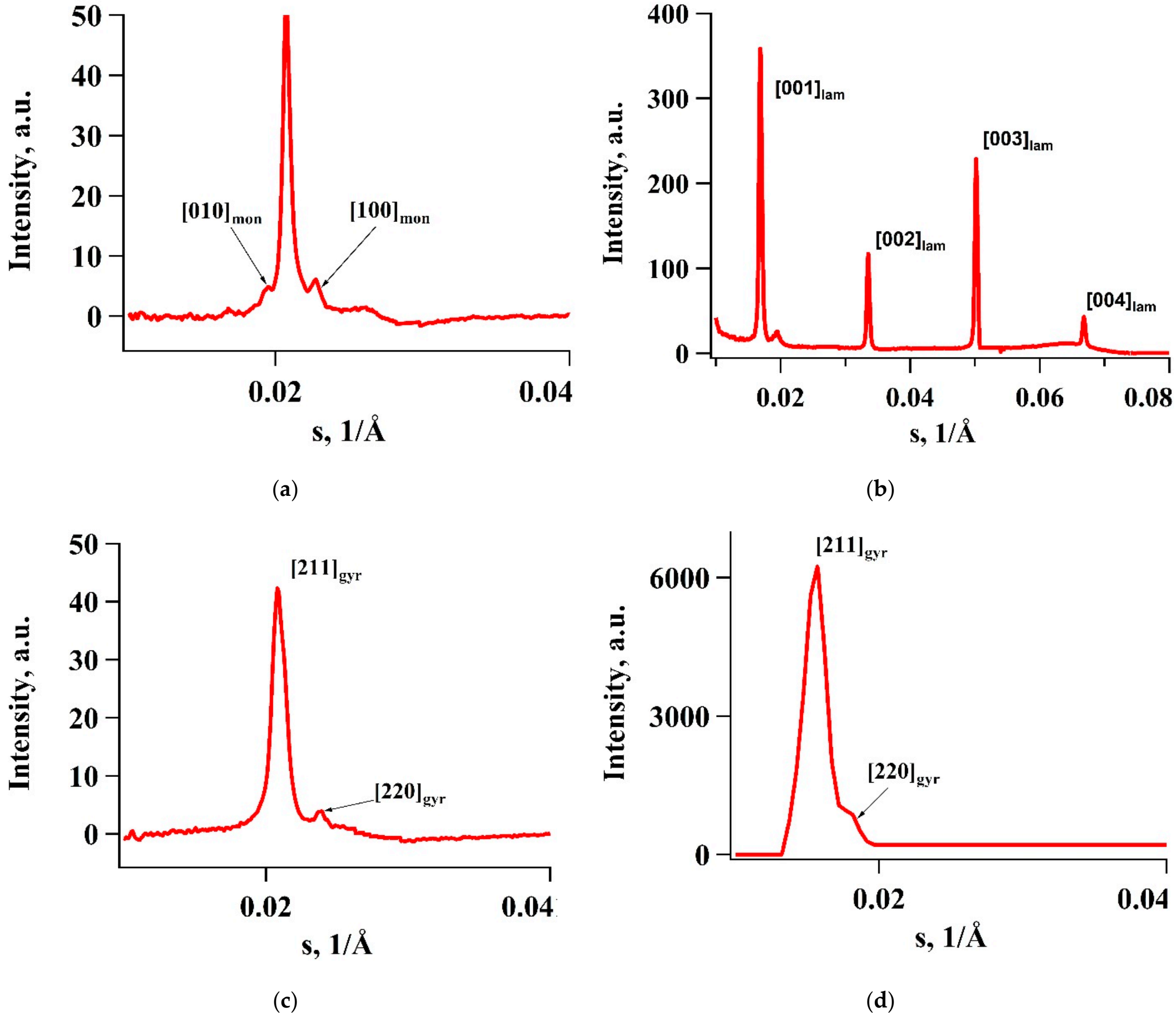

Figure S3. 1D-reduced GISAXS pattern of a dry C8AzoPyr thin film. (a) Columnar monoclinic (Col_{mon}) phase formed after long-term annealing at room temperature; (b) lamellar phase (lam) formed upon swelling in methanol vapors for two hours at room temperature; (c) double gyroid phase (Cub_{gyr}) generated after long-term annealing at ambient conditions; (d) Cub_{gyr} phase swollen in water vapors for two days at room temperature.

References

1. Grafskaia, K.N.; Anokhin, D.V.; Zimka, B.I.; Izdelieva, I.A.; Zhu, X.; Ivanov, D.A. An "on–off" switchable cubic phase with exceptional thermal stability and water sorption capacity. *Chem. Comm.* **2017**, *53*, 13217-13220.
